# Early Diagnosis in Cancer of the Tongue

**Published:** 1903-02-21

**Authors:** 


### EARLY DIAGNOSIS IN CANCER OF THE TONGUE.

In a clinical lecture delivered at St. Bartholomew's Hospital, and reported in the *British Medical Journal*, Mr. Butlin again urges the extreme importance of early diagnosis in cases of cancer of the tongue. He directs special attention to certain conditions of the tongue which are to be regarded as "predisposing causes," and others which are definitely "pre-cancerous conditions," and asserts that there is no internal organ of the body in which the early diagnosis of cancer is so easy as in the tongue. Nor ought it to be overlooked, seeing the frequency with which doctors, and even patients themselves, look at the tongue. In regard to predisposing causes, Mr. Butlin mentions several local conditions. There is a condition of chronic superficial glossitis with red patches on the dorsum and borders of the tongue, and on further examination cracks, fissures, excoriations, and chronic ulcers will be found. In another variety the tongue looks at first to be covered with bluish fur, a sort of film, perfectly smooth. There are no appendages or papillæ on it at all. In another there is what looks as if a thick daub of white paint had been placed upon the middle of the tongue. In another there may be an association of chronic superficial glossitis with this white material, which is called leucoplakia. Now these conditions are not themselves cancerous, but they are predisposing to such an extent that Mr. Butlin believes that if one took 100 cases of cancer of the tongue, in about 90 of them the cancer would have been preceded by one or other, or a combination of these conditions.

Whether these conditions of chronic superficial glossitis and its results be due to past syphilis, or rheumatism or gout, or to much smoking or much drink is of small consequence, the great thing is that people who have them are much more liable to cancer of the tongue than other people are. Moreover, when these conditions have once become established the tongue can never again be made like it was before; so that these conditions when they have once occurred retain their potency as predisposing causes of cancer throughout the patient's life. In regard to the precancerous conditions—conditions which lead up from one of the states just described to the actual existence of cancer, Mr. Butlin says there are certain conditions which are so likely to pass into cancer that they may be regarded as absolutely precancerous conditions. Warts and warty growths scattered about the tongue is the most common form in which cancer begins in these tongues.

Again, underneath the border of a tongue which is not in the least predisposed to cancer, a flat ulcer may form which may be ascribed to the rubbing of a tooth. But when the tooth is taken out the ulcer, instead of getting better, may slowly spread on the surface of the border of the tongue, its edges becoming a little raised and a little hard. This is a dangerous condition which is almost certain to become cancerous.

Again, in some of the white plaques before mentioned a small or larger area becomes thicker and a little more prominent and then tends to break down and soften in the centre, and out of that condition cancer will almost certainly develop. As to lumps and nodules in the substance of the tongue, and the old chronic ulcerations of the tongue, they rarely become cancerous; they are not nearly so dangerous as the indolent ulcer which makes steady progress in the course of a few weeks or months. You will ask, says Mr. Butlin, why it is that medical men watch these dangerous conditions for weeks and months until the cancer is absolutely established? Why do they treat them with nitrate of silver, and washes, and lotions, and dose the patient with potassium iodide, when cancer is developing under their eyes? The predisposing conditions of cancer of the tongue are as well-known as anything in surgery, while cancer begins on these predisposed tongues in such a way that anybody and everybody can see it, yet it is allowed to drift until too late.

Mr. Butlin says that if consulted about warts and warty growths, particularly on tongues, which are the seat of one of the "predisposing conditions," one should see that no time is lost in removing them by clean eliptical incisions down into the muscle. The wound being brought together will heal in a few days. Then in regard to indolent ulcers ascribed to the rubbing of a tooth, let the cause of irritation be removed at once, and if at the end of a week or ten days from that time the ulcer is not well or healing rapidly it also should be cut out in precisely the same fashion. No time is to be wasted.

Again, if white patches become thicker and a little more prominent, particularly if they show a tendency to break down in the centre, or soften, they also should be cut out. Indeed, in bad cases of superficial glossitis, where the patient is always suffering and always in dread of the onset of cancer, it may be desirable to have that portion of the tongue removed even when there is as yet no sign either of cancer or of the pre-cancerous condition.

